# PKCε Signalling Activates ERK1/2, and Regulates Aggrecan, ADAMTS5, and miR377 Gene Expression in Human Nucleus Pulposus Cells

**DOI:** 10.1371/journal.pone.0082045

**Published:** 2013-11-28

**Authors:** Emmanouella Tsirimonaki, Constantinos Fedonidis, Spiros G. Pneumaticos, Adamantios A. Tragas, Ioannis Michalopoulos, Dimitra Mangoura

**Affiliations:** 1 Biomedical Research Foundation of the Academy of Athens, Athens, Greece; 2 Department of Orthopedics, Athens Medical School, University of Athens, Athens, Greece; National Centre for Scientific Research, 'Demokritos', Greece

## Abstract

The protein kinase C (PKC) signaling, a major regulator of chondrocytic differentiation, has been also implicated in pathological extracellular matrix remodeling, and here we investigate the mechanism of PKCε-dependent regulation of the chondrocytic phenotype in human nucleus pulposus (NP) cells derived from herniated disks. NP cells from each donor were successfully propagated for 25+ culture passages, with remarkable tolerance to repeated freeze-and-thaw cycles throughout long-term culturing. More specifically, after an initial downregulation of *COL2A1*, a stable chondrocytic phenotype was attested by the levels of mRNA expression for aggrecan, biglycan, fibromodulin, and lumican, while higher expression of SOX-trio and Patched-1 witnessed further differentiation potential. NP cells in culture also exhibited a stable molecular profile of PKC isoforms: throughout patient samples and passages, mRNAs for PKC α, δ, ε, ζ, η, ι, and µ were steadily detected, whereas β, γ, and θ were not. Focusing on the signalling of PKCε, an isoform that may confer protection against degeneration, we found that activation with the PKCε-specific activator small peptide ψεRACK led sequentially to a prolonged activation of ERK1/2, increased abundance of the early gene products ATF, CREB1, and Fos with concurrent silencing of transcription for Ki67, and increases in mRNA expression for aggrecan. More importantly, ψεRACK induced upregulation of hsa-miR-377 expression, coupled to decreases in *ADAMTS5* and cleaved aggrecan. Therefore, PKCε activation in late passage NP cells may represent a molecular basis for aggrecan availability, as part of an PKCε/ERK/CREB/AP-1-dependent transcriptional program that includes upregulation of both chondrogenic genes and microRNAs. Moreover, this pathway should be considered as a target for understanding the molecular mechanism of IVD degeneration and for therapeutic restoration of degenerated disks.

## Introduction

A principal cause of lower back pain is degeneration of the intervertebral disc (IVD), a complex disease associated with specific environmental stressors and some genotypes [1]. The disk consists of the central gelatinous nucleus pulposus (NP) core and the external annulus fibrosus (AF) striated fibrocartilage that is attached through the endplates onto the vertebrae. The disk is a complex structure highly specialized for mechanical functions such as spine connectivity, flexure, rotation, and extension. Most of these functions are essentially attributed to the rich extracellular matrix (ECM) of the NP that the small and abundant early in life, chondrocyte-like NP cells produce. IVD degeneration is characterized and may be preceded by decreases in the production of proteoglycans, and in particular aggrecan, and type II collagen [2]. Replacing the traditional therapeutic approaches with those restoring disc properties has been a major challenge. Several studies have focused on the use of autologous cell implantation approaches in an attempt to regenerate the NP. Indeed, several cell sources, including mesenchymal stem cells from the bone marrow or the adipose tissue as well as NP cells, may be propagated in culture; these cells have been used for transplantation into animal models with promising results [3-6]. A second line of investigations has focused on soluble factors that may be used for therapeutic injections [7,8], should these factors and the biological processes that may control are identified and characterized. 

 Elucidation of the mechanisms that regulate the NP cell survival and differentiation would greatly advance all therapeutic efforts [9]. Deriving their lineage from notochord, an embryonic structure of mixed origin, NP cells possess features of chondrocytes [10,11], and, in animal species that retain notochordal cells in adult life, may benefit from paracrine signaling [12] or a progenitor-and-derivative relation [13]. Yet, the molecular basis of intercellular or intracellular signalling pathways that control the transcriptional programs of proliferation and differentiation in NP cells remains uncharted. Such programs are expected to entail delicate control of the expression of genes that regulate ECM composition, and in particular aggrecan, and those that degrade it, like ADAM metallopeptidase with thrombospondin type 1 motif, 5 (ADAMTS-5), the main aggrecanase in human NP [14] that is thought to promote IVD degeneration [15]. The study of these mechanisms has been aided by the recent availability of adequate cell numbers, obtained through culturing and subculturing of human nucleus pulposus cells, in their naïve state [7,11,16-20] or after immortalization with stable transfection of recombinant human telomerase reverse transcriptase [21], and key regulators have begun to be identified.

 Protein Kinase C (PKC), a family of serine/threonine protein kinases, has emerged as a key regulator of signaling pathways controlling proliferation, differentiation, and survival in the chondrogenic cell lineages. PKCs transduce extracellular signals from membrane receptors to the nucleus, and execute their regulatory roles acutely, by modifying through phosphorylation the function of protein substrates, i.e., other signalling, cytoskeleton and secretion-related proteins, as well as in the long-term, through MAPK activation, and thus activation of transcription factors and phenotypic gene regulation (e.g., 22-25). In addition, PKCs may mediate cross-talk with other major signalling pathways, including the PKA, wnt canonical and non-canonical, and the tyrosine kinase receptors pathway [26-29]. Most recently, a novel mechanism of action for PKC has been documented in the regulation of expression of microRNAs (miRNAs). These 20–25-nucleotide-long, endogenous, non-coding RNAs can target gene expression through translational repression and/or target mRNA for degradation in a sequence-dependent manner [30]. miRNAs most often respond to transcriptional activity that follows activation of signalling pathways, as part of transcriptional program regulation [31,32], and thus co-regulate major biological pathways with potential clinical uses (e.g., 33). 

 Aside the human nucleus pulposus cells where expression of PKC isoforms has not been addressed, many studies have dealt with the role of individual PKCs in all basic cellular processes of chondrocytes, including its action as a major signalling effector of activated vitamin D receptors [34], yet there is no clear consensus on specific mechanisms. The difficulty in delineating the roles of individual PKCs primarily lies with the fact that isoform-specific activators and inhibitors have only very recently been developed [35-37], while long term effects of PKC genetic manipulation usually include inter-regulation amongst the isoforms. Thus, in studies used in culture models activities of PKCα and ζ are reduced during nitric oxide-induced apoptosis in chondrocytes [38,39]; their role in dedifferentiation, however, appears as necessary [40,41] or indifferent [38]. In human articular chondrocytes cultures, PKCζ is required for cytokine-mediated expression of aggrecanases, a hallmark of osteoarthritis [42,43] and of metalloproteinases (MMPs), an irreversible mechanism of cartilage collagenolysis during inflammatory joint diseases [44]. Similarly, PKCδ is shown as necessary for chondrocytic survival [45], but, when activated by basic fibroblast growth factor (bFGF), stimulates MMP activity [46]. The novel PKCε has been detected in all tested chondrocytic lineages across species [40,45-47], yet its specific roles have to be addressed. PKCε was not found implicated in reactive oxygen species-induced chondrocyte cell death [45], while its activation was reduced by bFGF, a “catabolic” agonist in human articular chondrocytes [46]. Further pointing out a protective role, PKCε has not been detected in screens that identified genes upregulated in diseases of chondrocytes, like for example PKCβI [45], and ζ or ι [42,44]. Moreover, PKCε does not appear to participate in crosstalks between major biological pathways in chondrocytes, like PKCα [29,48] and ζ [49]. The study for the specific actions of PKCε has become possible with the development of a rationally designed peptide, namely ψεRACK, an PKC allosteric activator derived from a sequence that regulates autoinhibitory interactions [35,37]. ψεRACK, conjugated to TAT peptide, has been administered in culture and in vivo with equal success in producing PKCε specific biological effects [24,36]. With this background, we undertook these studies to investigate the mechanistic details of PKCε activation and study its potential function as a chondrocytic phenotype regulator in human nucleus pulposus cells. 

## Materials and Methods

### Materials

Culture media and supplements were from Life Technologies and fetal bovine serum (FBS) from Biowest. We used the following antibodies (and dilutions): polyclonal antibodies to tropomyocin (1:400) from Sigma, to phosphorylated species of PKCε (1:1,000) and myristoylated alanine-rich C-kinase substrate (MARCKS) (1:1,000), to PKCε (1:1,000), ERK1/2 (1:2,000), and to activator protein 1 (AP-1) (1:1,000) from Santa Cruz, to cAMP responsive element binding protein 1(CREB1) (1:1,000) from Millipore; mouse monoclonal antibodies to MARCKS (1:1,000) and phosho-ERK1/2 (1:2,000) from Santa Cruz, to p120GAP (1:2,000) from Upstate Biotechnology, to neo-epitope ARG on aggrecan, clone BC-3 (1:1,000 for Western blotting and 1:200 for immunocytochemistry) from ABCAM, and to β-tubulin (1:500) from Sigma; and secondary antibodies conjugated to horseradish peroxidase or fluorochromes from Santa Cruz. The PKCε-selective activator peptide, ψεRACK [PKCε (85-92), HDAPIGYD] [37] was synthesized and conjugated to a peptide derived from the trans-activating transcriptional activator (TAT) (amino acids 45-57, YGRKKRRQRRR) by Peptide 2.0 Inc. PD98059, U0126, and SB203580 were from Calbiochem.

### Human samples, isolation and culture of human nucleus pulposus cells

Human tissue samples of lumbar pathological IVD (Thompson grade 3 or 4) were donated from consenting patients (14 males and 6 females, aged 27 to 45) undergoing surgery for removal of herniated discs. The IVDs were maintained into DMEM:F12 and the nucleus pulposus was surgically dissected from the annulus fibrosus using a stereoscope. One tenth of each disk was acutely frozen in Trizol and the rest was used to establish primary cell cultures. Briefly, nucleus pulposus cells were released after mincing with scalpels and an overnight treatment with 0.025% collagenase (Worthington) at 37°C. Tissues were triturated with a pasteur pipette and debris removed with a 500 x g x1 min centrifugation; the resulting supernatant was washed and resuspended in DMEM:F12 1:1, supplemented with 10% FBS, 66μM L-ascorbic acid [50], 1mM L-Glutamine, 1% Gentamicin, and 1% penicillin/streptomycin. After plating onto culture dishes (1 IVD/60mm), cells were grown at 37°C and 5% CO_2_. Culturing media was replenished every 2-3 days and, when confluent, NP cells were trypsinized, counted, and split at a ratio of 1:3. Every 3-4 passages, some cells were frozen and stored in FBS:DMSO:glycerol:DMEM at a ratio of 50:10:10:30 at -90°C or -180°C, and were thawed with a similar success rate of 80% of the trials (maximum freezing time was 2 years). Articular knee cartilages (AC) were donated from three patients with trauma and no history of joint disease and were cultured as previously described [51]. 

### Ethics statement

For our studies, herniated disks from patients undergoing discectomy surgery for degenerative disc disease or knee cartilage shreds, removed from frayed cartilage edges during repair surgery for acute trauma, were obtained only from individuals that had previously given their verbal informed consent to release for medical research the tissue byproducts of their procedure. The oral consent was documented by an appropriate witness, following the protocol approved by the Ethics Committee, KAT Hospital, University of Athens. This protocol fully conformed to the ethical guidelines of the 1975 Declaration of Helsinki for Medical Research, including randomly assigned numbers to protect the anonymity of the patient.

### Immunocytochemistry, deconvolution microscopy, and Western blotting

Cell fixation and immunofluorescence analysis was performed as previously described (e.g., 23,52,53). For aggrecan detection, cells were treated postfixation with 5x10^-5^ U /µL of Chondroitinase ABC (Sigma) for 3 hours at 37°C. The primary antibodies were detected with species-specific rhodamine- or FITC-conjugated secondary antibodies as indicated in figure legends, the nuclear DNA with Hoechst 33258 (Pharmingen), and F-actin with Alexa Fluor 633-phalloidin- (Invitrogen). Cell staining was visualized using an Axiovert 200M inverted fluorescence microscope with a motorized stage and a Hamamatsu Orca-ER CCD camera; images from double or triple staining were obtained at the same focal planes of serial 0.5μm thick Z dimension optical sections and resulting Z-stacks were deconvolved with Slidebook™.

 Immunoblotting, using total cell homogenates in RIPA buffer or concentrated, chondroitinase-treated culture media were performed as previously described (e.g., 23,53-55). Twenty five µg of protein or volumes corresponding to 500 µg of cellular protein were analyzed per condition. Following separation with SDS polyacrylamide gel electrophoresis, proteins were transferred to nitrocellulose membranes, and analyzed by indicated antibodies and the appropriate species specific secondary antibodies conjugated to horseradish peroxidase. Immunoreactivity was visualized by enhanced chemiluminescence (Santa Cruz). Chemiluminescence detection was linear at 30-60 sec of exposure for all antibodies used except for BC-3 which was 5 min. 

### RT-PCR analysis

Semi-quantitative PCR was performed as previously described [22,56]. Briefly, total RNA isolated from NP, AC, and 90-8 (a malignant peripheral nerve sheath tumor, MPNST, cell line) cultured cells using the Trizol reagent (Ambion) was reverse transcribed (1 µg per reaction) with SuperScript II reverse transcriptase (Invitrogen), and 1µL of the formed cDNA was amplified with Taq DNA Polymerase (Hytest), in the presence of gene-specific primers for: *ACAN* F GAGAAGTTCACCTTCCAG GAAG, R CTGCACATGTCCATGCCA; *ADAMTS5* F GAGGATTTATGTGGGCAT CATTCATGTG, R CATATGGTCCCAACGTCTGC; *SOX5* F CAAGGCAATC ATGCGCAACA, R TGCTAGACACGCTTGAGTGC; *SOX6* F GATGCCA TCAACTCCACAGC, R GCTGCAGAGCCATTCATTGC; *SOX9* F ACAA GAAAGACCACCCCGAATAC, R TGCTCAGTTCACCGATGTCCAC; *COL2A1* F TTCAGCTATGGAGATGACAATC, R AGAGTCCTAGAGTGACTGAG; *BGN* F TGCAGAACAACGACATCTCCG, R GGAGGAGCTTGAGGTCTGGGA; *LUM* F AAGGCCTTTGAGAATGTAACTG, R TGTTGCTGATCTTATTGTTGTCTA; *FMOD* F ACCGTCCCCGATAGCTACTT, R CATCCTGGACCTTCCAGCAAA; *PTCH1* F GAAGGTGCTAATGTCCTGAC, R GTGCTGTTCTTGACTGTGCC; *ki67* F ACCCTGCGACTCTCCACAGT, R GCTCCTCTGTACGTCCCTTTT; and *GAPDH* F ATGGTGAAAGTCGGAGTCAA, R ATCACAAGTTTCCCGTTCTC. Primers for the PKC genes were as in Oshevski et al. 1999 [57]. Small RNAs were isolated using the MIRVANA kit (Invitrogen) and Homo sapiens (*hsa*)*-miR-377* or the mRNA for the *U6* were detected using a specific stem-loop adaptor primer set F GCCGCATCACACAA AGGCAAC, R GTGCAGGGTCCGAGGT, and primers F CTCGCTTCGGC AGCACA, R AACGCTTCACGAATTTGCGT, respectively. 

Amplification conditions were an initial denaturation step of 4 min at 94°C followed by 33-40 cycles (linear region) at 94°C for 30 sec, at 52°C - 62 °C (depending on annealing temperature of each primer set) for 30 sec, at 72°C for 30 sec, followed by 10 min at 72°C. PCR products, separated in appropriate for each product percentage agarose gels, were stained with ethidium bromide and visualized under UV light using the Dolphin-Doc Pro system. For quantification, band intensities were assessed using the ImageJ software and normalized in reference to controls *GAPDH* (genes) or *U6* (*miRNAs*), which were always amplified for each cDNA preparation in their linear region of 23 cycles. 

### Databases

*In silico* analysis of *ADAMTS5* 3’-UTR to identify predicted binding sites of microRNA was performed using the following databases: TargetScan Release 6.2. (http://www.targetscan.org), microrna.org (http://www.microrna.org ), miRDB (http://mirdb.org), and DIANA-microT 3.0 (http://diana.cslab.ece.ntua.gr/microT). Genomic sequences for pairwise alignments were downloaded from EnsEMBL (http://www.ensembl.org) and AP-1 and CREB1 transcription binding sites were identified using the ConTra web tool (http://bioit.dmbr.ugent.be/ConTra/index.php) as previously described [58-62].

### Statistical analysis

Experiments were performed 2-5 times and numerical data were analyzed by ANOVA; a P value of less than 0.05 was considered significant.

## Results

### Establishment and characterization of human NP cell cultures of early and late passages

**Figure 1:**
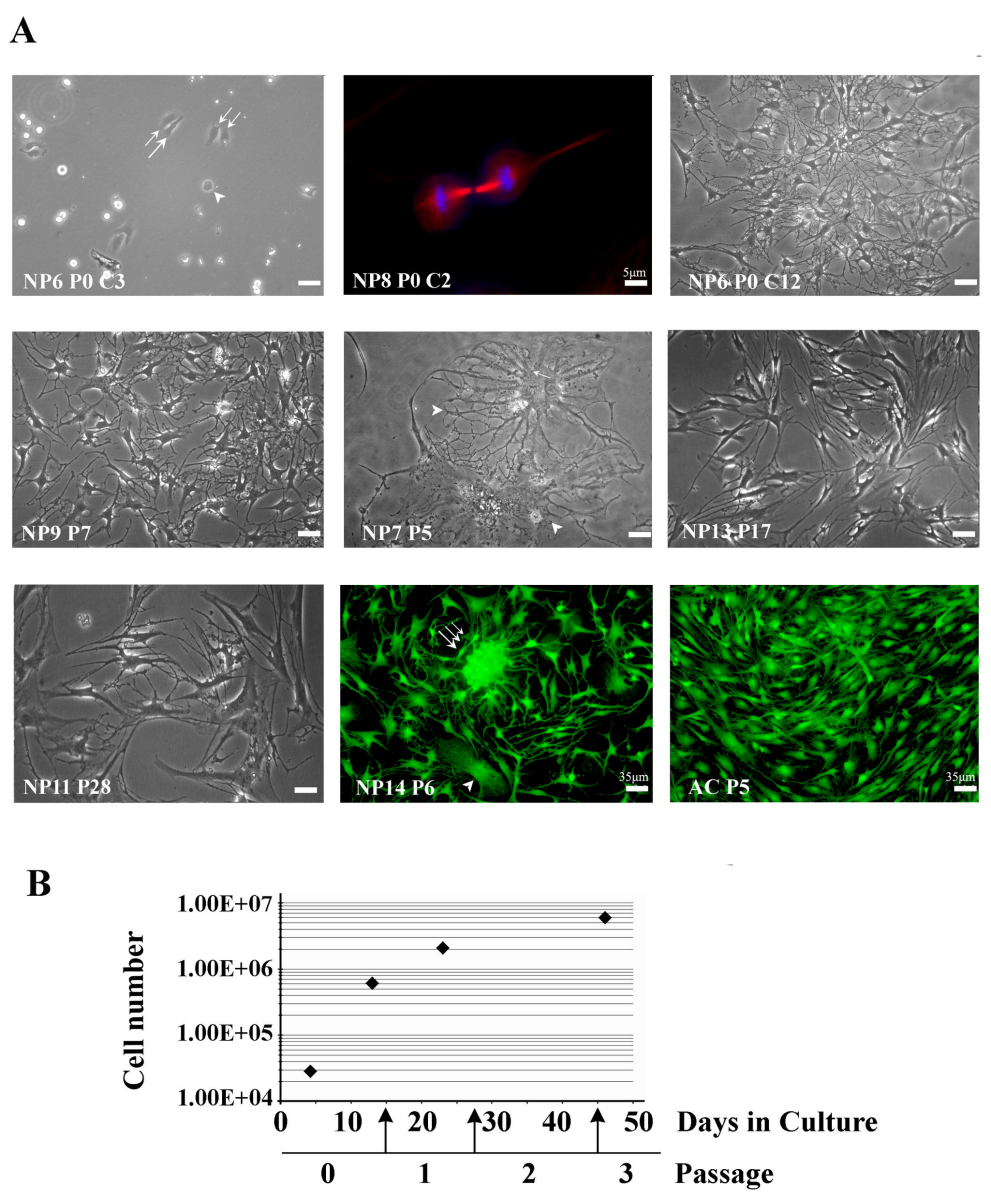
Morphologies and growth rate of human NP cells in culture. (**A**) Phase contrast micrographs (black-and-white panels), and immunocytochemical decoration of mitotic spindles (microtubules visualized with β-tubulin-rhodamine and chromosomes with Hoechst 33258, upper row middle panel) and of the actin-binding protein tropomyosin (visualized with fluorescein, last two panels) show gains in numbers and a stable morphology throughout patients samples and passaging, even after freeze-thawing cycles (examples are given for samples NP6, 9, 11, 13, and 14; arrows and arrowheads are described in the text). NP or AC cells grew without contact inhibition, while NP cells could form cellular hives in early passages (triple arrow in NP14 P6). Large NP cells, accounting for ~1%, were observed in all samples (arrowheads in NP7 P5 and in NP14 P6); bar=10 μm in phase contrast photographs. (**B**) Cell number (logarithmic scale, y-axis ) versus days in culture (linear scale, x-axis): NP cells had impressive growth rates in early passages with similar curves amongst patient samples (data shown are the average of all samples); arrows indicate times of cell culture propagation through passaging.

In order to attain sufficient NP cell numbers to study mechanisms of differentiation, we reasoned that NP cells should be allowed to first partially de-differentiate and thus gain in proliferation capacity. In pilot experiments we established the conditions, described in Methods, which permitted both cell cultures from every patient to be established and propagation of these cultures for up to 30 passages. When freshly plated, cells exhibited spherical shapes as they underwent cell division (Figure 1A, NP6 Passage 0 (P0), culture day 3 (C3), arrowhead, and NP8 P0, C2), before spreading onto the substrate (double arrows, NP6 P0, C3). Over the next days, cells acquired a polygonal body with several processes radiating away from the cell body, thus assuming an irregular stellate appearance (Figure 1A, NP6, P0, C12); such NP cell morphology with several lamellipodia with filopodia has been previously reported for human (e.g., 16,63) or bovine NP cell cultures, while in cell cultures established from explants [11] or through mechanical dissociation (filters) [17] lesser lamellipodia, often without filopodia, were observed at least during early passages. Cells grew exponentially during the first few passages (Figure 1B), and proliferation rate remained high up to passages 10-12, even after propagation (repeated passages and cycles of freeze-and-thaw) that covered cell maintenance periods (culture plus storage) of over two years in total. Cell morphology was also very similar over different patient samples e.g., Figure 1A, NP6 P0 at C12, and NP9 P7, NP13 P17, and NP11 P28 (all at C10). It is noteworthy that also up to passage 10-12, about 1-2% of the cell population consisted of large body cells with intricate process outgrowth patterns (Figure 1A, arrowheads in NP7 P5 and NP14 P6). Beyond that, cell proliferation slowed down, yet cells kept their stellate appearance and responded to plating on chitosan with a rounded morphology (data not shown). In general, cells grew without contact inhibition and we often observed cellular hives (Figure 1A, triple arrow) at least up to P10. Contact inhibition was also absent in cultures of human articular chondrocytes (AC), which were used as reference of a chondrocytic phenotype; these cells, however, grew without hive formation (Figure 1A, AC P5). 

**Figure 2:**
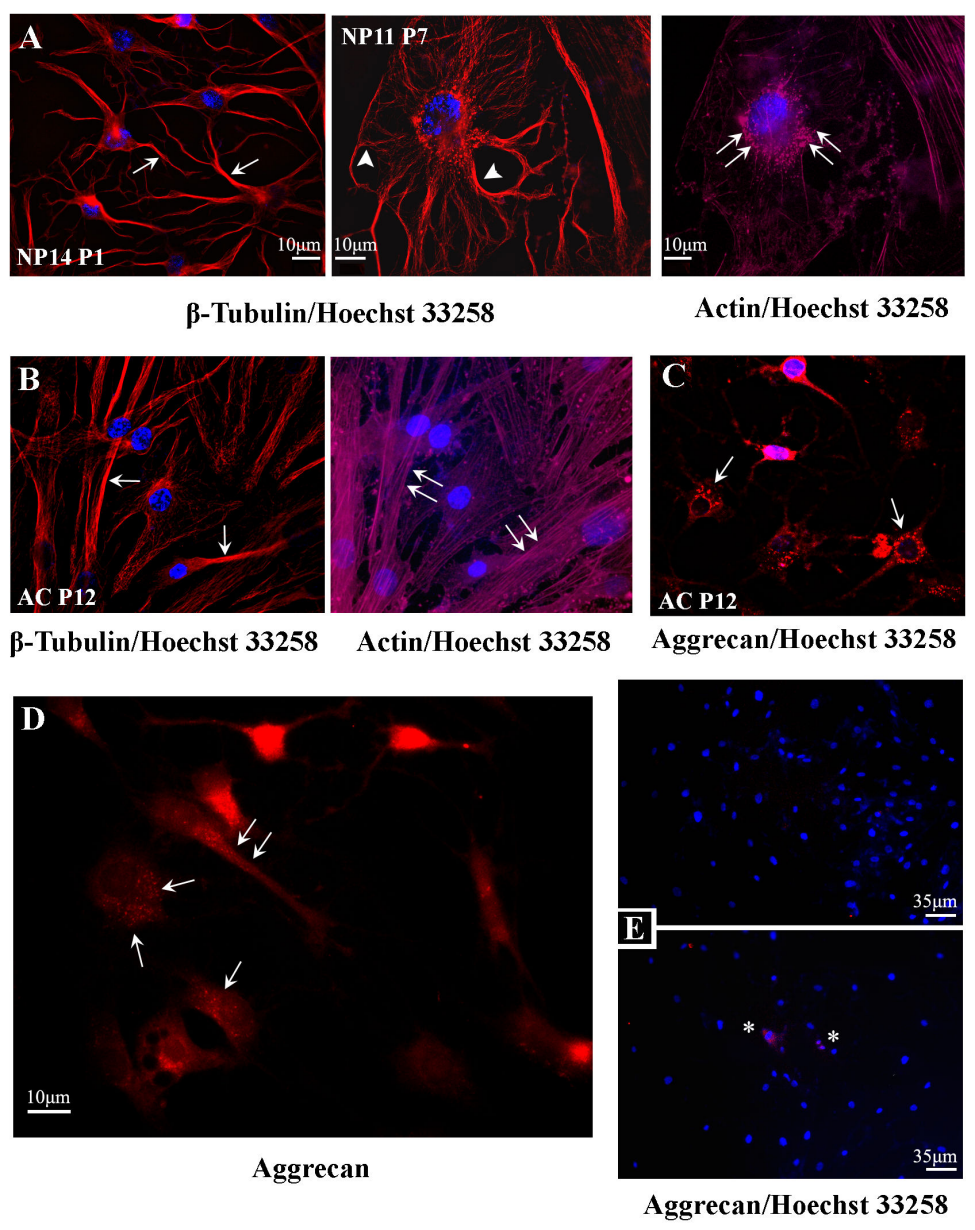
Cytoskeletal and chondrocyte phenotypic markers in cultured NP cells. NP cell cultures were fixed, immunostained and imaged with deconvolution microscopy (**A**) β-tubulin immunostaining (red) reveals elaborate microtubule cytoskeletons in NP cells (arrows), especially in the rare large notochordal-like cells (arrowheads), while phalloidin staining (magenta) shows that F-actin is organized in focal adhesions under the cell body (double arrows); nuclei are stained blue with Hoechst. (**B**) In contrast, AC cells exhibit polarized cell bodies, lesser microtubule branching (arrows), and F-actin forms intense stress fibers (double arrows). Staining with the BC-3 antibody (red) that recognizes the neoepitope on human aggrecan that is created after cleavage within the sequence TEGE373-_374_ARGS by aggrecanases activity, decorated the cytoplasm of both (**C**) AC cells and (**D**) NP cells. In both cell types the staining pattern was consistent with a vesicular distribution (arrows). (**E**) Aggrecan staining was not detected when chondroitinase was omitted (upper panel) or when the reaction was allowed to proceed at 4° C (asterisk indicate cells at mitosis) (NP6 P10, C7). NP tissue indicate fresh tissue sample, NP numbers indicate patient sample, and P the culture passage; all cells were visualized with a 63x oil immersion lens.

 NP cells exhibited elaborate cytoskeletons of microtubules and F-actin as observed with deconvolution microscopy and immunostaining with antibodies to β-tubulin and staining with phalloidin-Alexa Fluor 633, respectively (Figure 2 A and B). These patterns emphasized their ramified cytoplasm (Figure 2A, arrows), in particular of the large NP cells (Figure 2A, arrowheads), as compared to AC cells, which kept a triangular or bipolar cell body with fewer processes and lesser microtubule branching (Figure 2B, arrows). We observed little cell body motility in live microscopy (data not shown), while the phalloidin patterns revealed that most F-actin was organized in focal adhesions in NP cells but in stress fibers in AC cells (double arrows in Figure 2A versus Figure 2B). Deconvolution microscopy revealed that aggrecan immunostaining, with an antibody that recognizes ADAMTS-cleaved aggrecan, decorated the cytosol of all NP cells in a vesicle-like pattern, in particular around the nucleus and along major processes (arrows in Figure 2D), reminiscent of Golgi exocytic vesicles and ADAMTS-5 intracellular staining patterns [64,65]. The specificity of the staining was confirmed when omission of chondroitinase (Figure 2E upper panel), or incubation at 4°C (Figure 2E lower panel) dissipated staining, except in cells that were dividing and the nuclear lamina was dissolved (asterisks). Thus, BC-3 staining was feasibly the result of post-fixation activity of an aggrecanase, while we may not exclude the possibility that this was internalization of shorter, _374_ARGS-led aggrecan fragments, internalized from the ECM for intracellular destruction [66]. 

**Figure 3:**
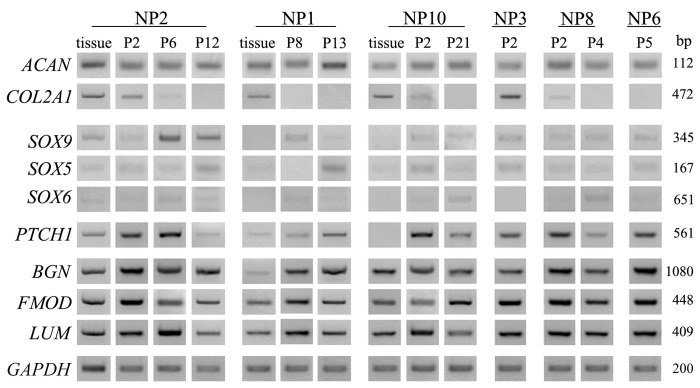
Chondrocytic marker expression remains stable over many cell passages of NP cell cultures. Representative images of RT-PCR results demonstrate expression levels of ACAN; COL2A1; *SOX* 5,6, and 9; PTCH1; *BGN*; FMOD; and *LUM* in NP tissue and in early and late passage of NP cells from different patient samples, and highlight that, under monolayer culture expansion, messages for ECM proteoglycans aggrecan, biglycan, fibromodulin, and lumican were sustained at similar levels throughout passaging. For each sample, the expression of each gene was examined using identical amounts of cDNA template, as for the housekeeping GAPDH (shown at the bottom panel).

 Cells of early or late passages expressed mRNAs for the most important component of the ECM of nucleus pulposus, the large chondroitin sulfate proteoglycan aggrecan (Figure 3). Moreover, at least message levels did not wither with cell plating or passaging, nor differed amongst samples (i.e. NP1 tissue versus P8, 13 or NP2 tissue versus P2, 6, or 12 or NP10 tissue versus P2 or P21). The mRNA expression for the chondrocyte marker type II collagen α1 was, however, downregulated, suggesting that these culture conditions promoted proliferation at the expense of differentiation. The mRNA levels for the three members of the SOX family of transcription factors, which are causally linked with the chondrocytic phenotype, namely SOX5, 6, and 9, were often higher in plated cells than the parental NP tissue (Figure 3). Interestingly, in NP tissues the mRNA expression for Patched-1 (*PTCH1*), the receptor for the chondrogenic Hedgehog proteins, was detected variably, yet in cultured cells it was steadily expressed. The mRNA expression patterns for the small proteoglycans biglycan, fibromodulin, and lumican were overall stable over long term culturing, including periods of freezing and reviving, and similar to the tissue levels (Figure 3), further indicating that NP cells remained committed to a stable chondrocytic phenotype. 

**Figure 4:**
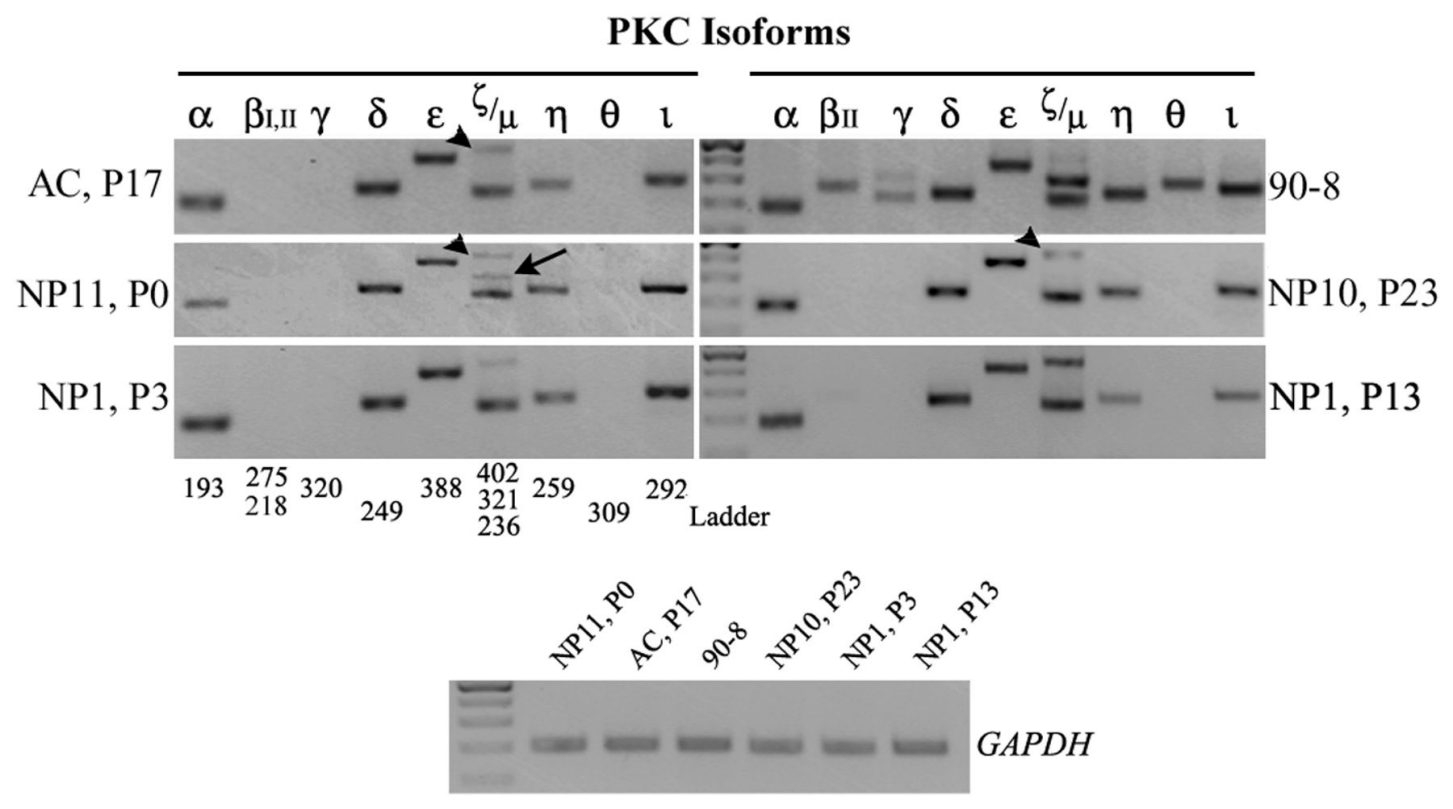
PKC isoform expression in human NP cells of early or late passage. Representative images of RT-PCR results for PKC isoform profiling demonstrate an indistinguishable profile amongst various NP samples and cell passages (NP11, P0; NP10, P23; NP1, at P3 and P13), and AC cells (AC, P17) in culture, with the exception of the ζ isoform, which appears at a slightly different molecular size (402 versus 321 - arrowheads versus arrows). The MPNST 90-8 cell line which expresses all the PKC isoforms but βI, served as a positive control. For each sample, the expression of each gene was examined using identical amounts of cDNA template, as for the housekeeping GAPDH (shown at the bottom row). The ladder used was the GeneRuler 100 bp (NEB), where the intense band corresponds to the 500 bp size.

 PKCs have important role in the proliferation, differentiation, or apoptosis of chondrocytes, yet, their molecular profile has not been previously addressed in human NP cells. Thus, we used semi-quantitative PCR to detect all known PKC isoforms in human NP-derived cultures, using as reference AC cells as well as the 90-8 cell line that expresses all PKCs, but βI (Figure 4). Throughout patient samples, mRNAs for PKC α, δ, ε, ζ, η, ι, and µ were steadily detected, whereas β, γ, and θ were not. This pattern was stable and was retained in all culture passages, either early (NP11, P0; NP1, P3) or late (NP10, P23; NP1, P13). An intriguing observation was that, upon subculturing, the ζ isoform consistently appeared at a slightly different molecular size (402 versus 321, arrowheads versus arrow), possibly due to alternative splicing, and this eventually became the prominent transcript. AC cells presented with an almost identical molecular PKC phenotype to that of NP cells, expressing the same isoforms at comparable ratios. 

### PKCε activation leads to gains in differentiation of NP cells

**Figure 5:**
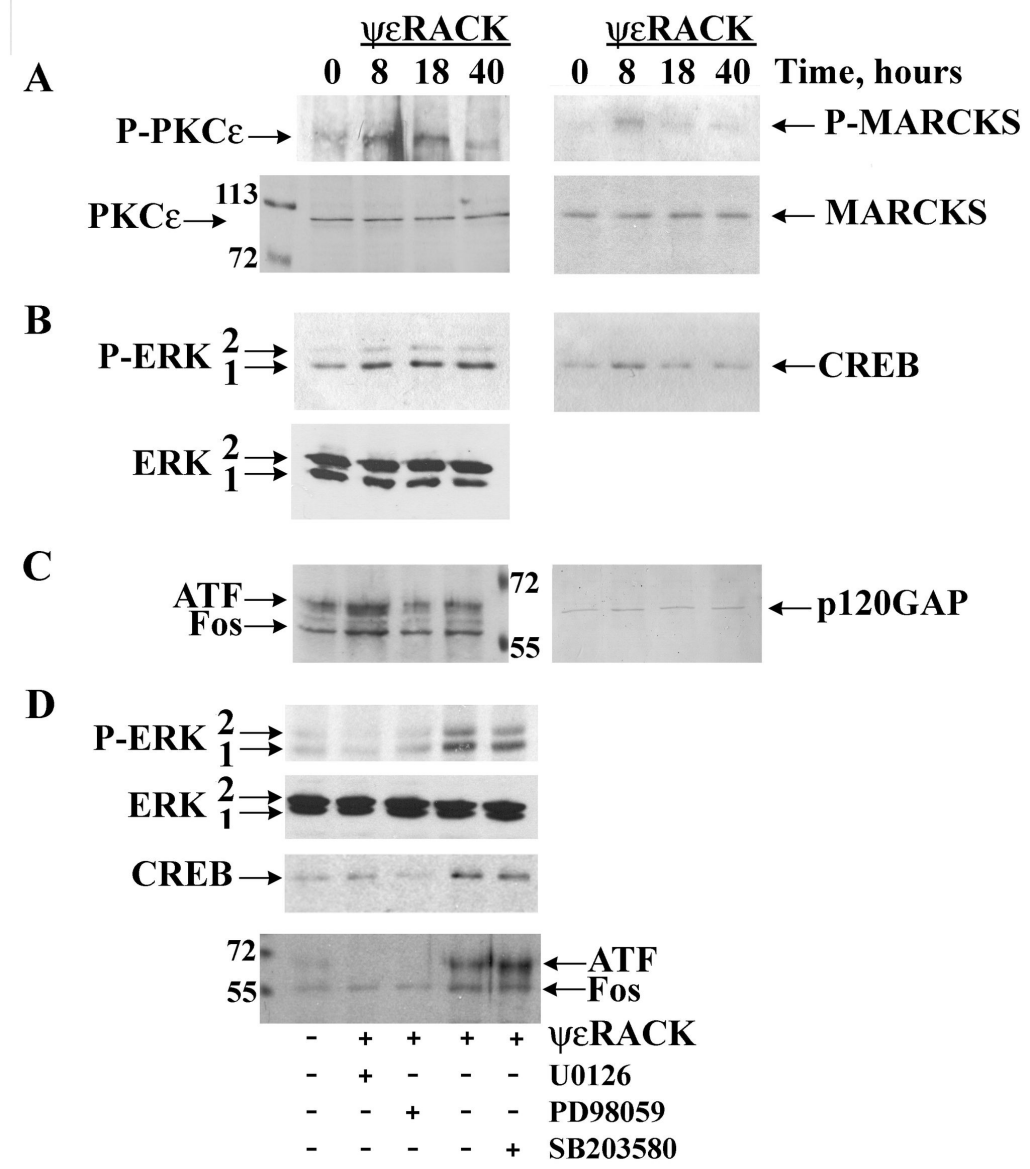
PKCε activates ERK and transcription factors AP-1 and CREB1 in human NP cells. Western blot analyses of NP cell lysates (25 µg of protein per lane) treated with vehicle (time 0) or with the PKCε-selective activator peptide ψεRACK (1μM) for 8, 18, or 40 hours show (**A**) ψεRACK-dependent increases in phosphorylation of PKCε (P-PKCε, left upper panel), and of the PKC-specific substrate MARCKS (P-MARCKS, right upper panel) (lower panels show PKCε and MARCKS abundance, respectively); (**B**) time course of PKCε-induced ERK1/2 phosphorylation, which establishes a moderate and sustained ERK activation up to 40 hours (left upper panel) (lower panel shows ERK1/2 protein abundance), and of PKCε activation-dependent increases in expression of the transcription factor CREB1 at 8 hours (right panel); (**C**) significant increases in expression of transcription factors Fos and ATF also at 8 hours after exposure to ψεRACK; equal loading for CREB1, Fos, and ATF was additionally monitored with immunodetection of p120GAP (right panel) on the upper part of the nitrocellulose membranes; (**D**) that ψεRACK-dependent ERK, CREB1, ATF and Fos activation was abolished by U0126 and PD98059, both inhibitors of MEK1/2, but not SB203580 (all inhibitors were added 20 min prior to treatments with ψεRACK). Similar results were obtained in five different NP cell lines; examples shown are from NP13 P8-P12 (A-C) and from NP11 P9-P13 (D).

In pilot experiments, we used expression levels of *SOX9*, which readily reflects initiation of transcription for chondrogenic differentiation, and a combination of isoform specific activators or inhibitors and antisense oligonucleotides to evaluate effects of individual PKC isoform and found that activation of PKCε with ψεRACK, a peptide that selectively binds to and activates PKCε, significantly increased expression of *SOX9* (Fig. S1). Thus, we investigated the signalling coupling of PKCε and ERK in NP cells. Using Western blot analyses we verified time-dependent phosphorylation/activation of PKCε after exposure to ψεRACK and phosphorylation of the PKC-specific substrate MARCKS (Figure 5A). Activated PKC is necessary for activation of the ERK cascade, and this was the case for PKCε activation in this chondrocytic cellular context which led to phosphorylation/activation of the MAPK ERK1/2 (Figure 5B), but not the MAPKs JNK or p38 (data not shown). Moreover, this PKCε/ERK pathway led to significant activation of transcription factors that are important for chondrogenic differentiation at 8 hours, namely, significantly increased expression of CREB1 (Figure 5B), and of activating transcription factor (ATF) and Fos (Figure 5C). These inductions were completely inhibited by U0126 (10 µM) and by PD98059 (20 µM) that prevent activation of MEK1/2, the upstream activator of the MAPK ERK1/2, whereas the selective inhibitor of the MAPK p38 SB203580 (10 µM) had no effect (Figure 5D). Taken together, these results attest for a central and specific role for PKCε in the activation of MEK1/2-ERK1/2 in NP cells, while its temporal mode suggested possible regulation, in addition to early genes CREB1 and AP-1, of late, or phenotypic gene transcription. 

**Figure 6:**
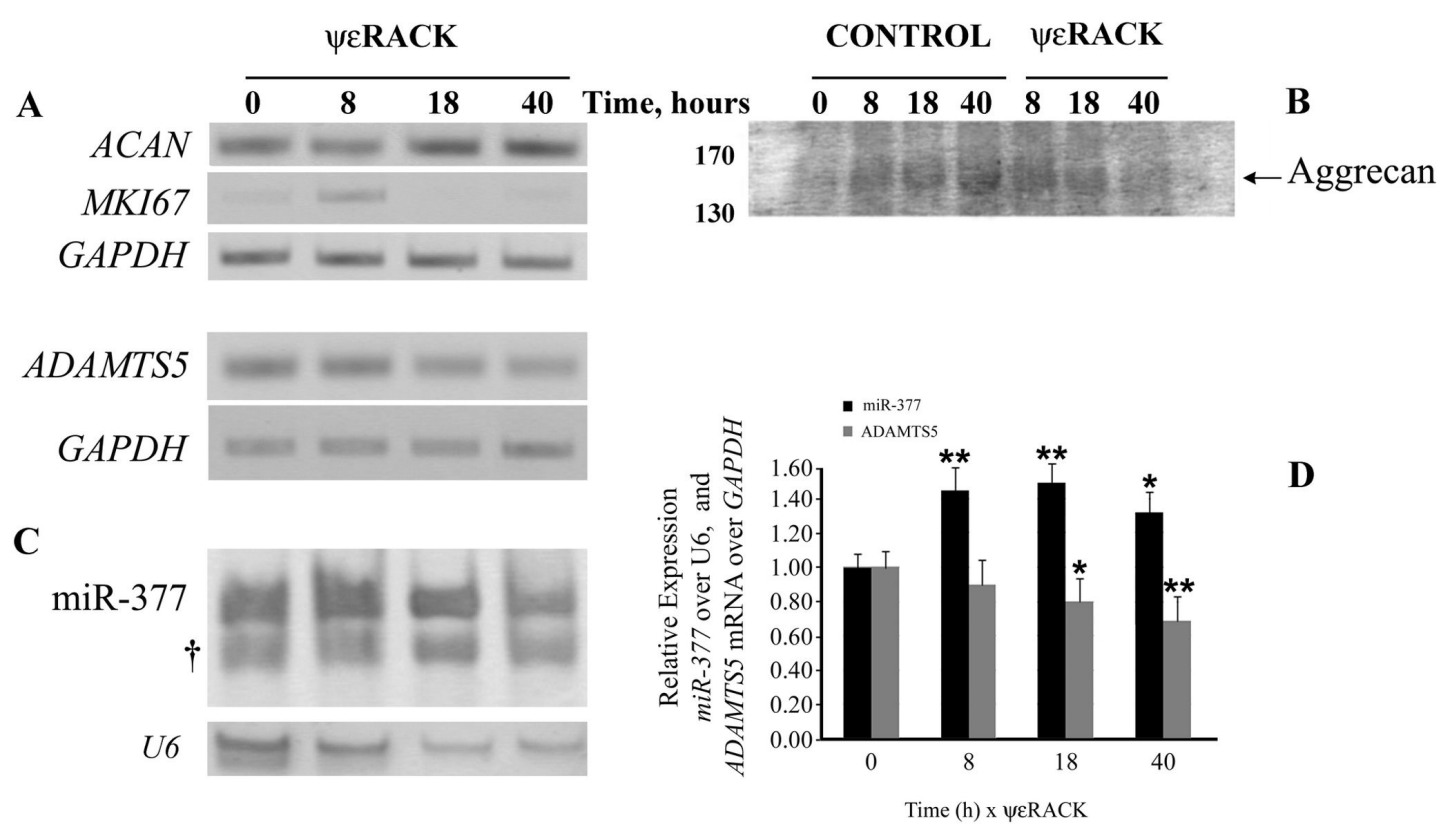
PKCε activation induces aggrecan gene expression, decreases ADAMTS5, and detection of ADAMTS-5-depended cleavage of ECM aggrecan through upregulation of hsa-miR-377 expression. (**A**) RT-PCR detected the relative expression of ACAN, MKI67, ADAMTS5 after treatment of NP cells with 1 μM ψεRACK (or vehicle) for indicated times and revealed that time-dependent activation of PKCε significantly increased the mRNA expression for aggrecan, while in an opposing manner decreased ADAMTS5; mRNA levels of the proliferation marker Ki67 became barely detectable after 8 hours of PKCε activation. (**B**) Immunoblotting of ECM proteins isolated from the culture media of NP cells with BC-3, an antibody that detects ADAMTS-5-mediated cleavage of human aggrecan, revealed a drastic decrease of cleaved aggrecan by 40 hours of treatment with ψεRACK, in agreement with the lower levels of ADAMTS5. (**C**) Levels of expression of hsa-miR-377, predicted to bind at several sites of ADAMTS5 3’-UTR, were significantly induced with PKCε activation, as determined with RT-PCR; † indicates the hsa-miR-377 primers. (**D**) Quantitation of hsa-miR-377 levels showed early sustained gains with ψεRACK (P < 0.01 at 8 and 18 h), whereas ADAMTS5 decreased gradually (P < 0.01 at 40 h); GAPDH and U6 were used for normalization of ADAMTS5 and hsa-miR-377, respectively. Bars in the graph represent the mean results of four independent experiments ±SEM, in which two different NP lines (NP6 P10 and NP8 P11 were run in parallel (n=8 for each time point), * P < 0.05 and ** P < 0.01 relative to 0 h ψεRACK.

 To investigate whether PKCε may promote phenotypic changes in NP cells, we examined the transcription of the aggrecan gene. PKCε activation had a remarkable positive effect on the transcription for aggrecan, as its mRNA, after a small reduction at 8 hours of exposure to ψεRACK, rose at 18 hours and remained high for at least up to 40 hours (Figure 6A, *ACAN*). Moreover, examination of the levels of the mRNA for the proliferation marker Ki67 (*MKI67*) revealed that, after an increase at 8 hours with ψεRACK, its expression became almost undetectable, indicating cell cycle withdrawal. When we assessed the gene expression for the major NP aggrecanase ADAMTS-5, we found that ψεRACK treatment readily and significantly downregulated the expression of *ADAMTS5*, with low levels persisting into 40 hours (Figure 6A). These decreases correlated flawlessly with the decreases in ADAMTS-cleaved aggrecan in the medium of NP cultures, as seen with immunodetection of the neoepitope ARG on aggrecan (Figure 6B). Moreover, both effects of ψεRACK on *ACAN* and *ADAMTS5* transcription were reversed with preincubation of the cells with U0126 (10 µM; 20 min ahead) (Figure S2), providing further evidence for their dependency on ERK activation. 

*ADAMTS5* transcription should have been upregulated, as expected from studies with overexpression of constitutively active CREB [67], we reasoned that this unexpected downregulation of ADAMTS-5 mRNA was due to microRNA activity. Our *in silico* analysis showed that the 3’-UTR of *ADAMTS5* holds an impressive 65% of the length of its mRNA (6141bp/9056bp), one of the longest amongst all known transcripts. Further bioinformatics surveys predicted that miR-377, a conserved amongst mammals miRNA, could bind on six sites (797-803, 1304-1332, 2937-2943, 3126-3133, 4775-4803, 6056-6062) on the 3’- UTR tail of *ADAMTS5*. To validate *ADAMTS5* as a target of miR-377 and as part of the transcriptional regulation imposed upon PKCε activation, we designed specific primers and amplified hsa-miR-377 in control and ψεRACK-treated NP cells. Fascinatingly, we found that PKCε activation upregulated the expression of hsa-miR-377 as by 8 hours of exposure to ψεRACK hsa-miR-377 levels had peaked significantly, and remained high thereafter (Figure 6C and graph in 6D). Our results suggest that PKCε activation increased the expression of hsa-miR-377, which led to long-term downregulation of *ADAMTS5*, causing in turn reduction in cleaved aggrecan in NP cells ECM. 

## Discussion

In order to understand the molecular basis of the differentiation process of human NP cells, we established monolayer culture conditions that would allow control over this event. These basic conditions led to some de-differentiation and significant gains in proliferation, which allowed us to passage cells many times, with cells retaining mRNA expression of chondrocytic markers, except for Col2A1, at similar levels to those detected in the corresponding parental tissue, as previously reported for rat NP cells after few weeks in culture [17,68,69]. Specifically, NP cells expressed detectable levels of *SOX9*, an important for the initiation of chondrocytic differentiation transcription factor [70], throughout culturing periods of over 20 passages. Moreover, SOX9 cooperatively with SOX5 and 6 maintains a chondrocytic phenotype by directly regulating the transcription for the two other classical markers of NP cells, aggrecan and type II collagen [71]. NP cells also steadily expressed high levels of aggrecan message, confirming and extending the observation that during monolayer expansion of NP cells, after a transitional reduction, aggrecan, but not Col2A1, levels may stabilize [17]. Apparently, once released from the tissue, the transcriptome at first responded to the new environment in culture, which includes a number of stimulators including normoxia and serum growth factors, such as lysophosphatidic acid (LPA), with intense proliferation (as in rat chondrocytes, [72]). This finding shows that the mitotic program is constantly suppressed in the disk as reported by others (e.g., 7,11,17,19,20); as, however, the transcriptome in parallel adopts to the new environment by reverting to a nodal yet proximal point of differentiation in the tissue, cells are led to differentiation, hence the expression of NP markers, which is then stabilized under constant, non-challenging culture conditions. Indeed LPA receptors, highly abundant in nucleus pulposus cells [9], signal through PKC and PI3K/Akt, to additionally promote survival and differentiation (in resting chondrocytes [73]). While we did not assess matrix synthesis, we should reiterate that the cell lines we developed showed tolerance to repeated freeze-and-thaw cycles in terms of general growth characteristics and molecular phenotypic features. Thus, our data highlight for the first time this aspect of the remarkable plasticity that the NP cells apparently possess. 

 Moreover, under these conditions we observed the survival and propagation for several passages of a subpopulation of large cells, with a body diameter over 30 µm (Figures 1A, and 2A), with morphological phenotypes resembling “physaliferous” notochordal cells [13]. This pool of cells survived for about 10 passages and as it dissipated, so did the high proliferation rate of the culture. It was unfeasible to study whether these cells transformed to the smaller stellate cells, it appears, however, that these cells supported proliferation, possibly through paracrine functions, for example secretion of growth factors. Recently, a pool of cells with notochordal lineage markers was discovered in the adult bovine caudal disc, previously thought to contain only chrondrocyte-like NP cells and novel markers have been proposed [74,75]. Based on these facts we speculate that these cells may represent a notochordal population in the NP believed to dissipate by the age of 10 in humans, possibly through maturation into NP cells [13]. 

 We then proceeded to identify signaling pathways that may regulate this molecularly stable phenotype, focusing on PKC. Many studies have addressed the function of PKCs signalling in chondrocytes, as elaborated earlier, yet PKC profiling has attracted little attention. With the current studies we postulate that NP cells in culture express mRNA for PKC isoforms α, δ, ε, ζ, η, ι, and µ but not for β, γ, or ϑ. In studies using rat NP cells in monolayer cultures, targeted investigation of PKCα, γ, ε, ζ, and ι expression identified mRNAs for all of these isoforms [26]. Differences in the expression of PKCs, in this case of γ, between rat and human should be expected, as the standing position of humans imposes through mechanotransduction a different and constant genetic pressure on gene transcription. A consideration may be that the NP cells in our study derived from symptomatic patients, yet, the human AC cells from healthy donors exhibited an identical PKC profile. AC cells may have different lineage, they do, however, fulfill similar functional purposes and share common chondrocytic characteristics with NP cells, therefore the PKC profile we now report may be a “blueprint” for the human chondrocytic phenotype. 

 When we specifically activated PKCε with ψεRACK to explore its actions on chondrocytic differentiation we made a series of novel observations. First we found that PKC phosphorylates *in vivo* MARCKS, an F-actin binding protein that has been identified as a new therapeutic target of antirheumatic drugs and possibly a marker for cartilage/chondrocyte integrity and regeneration of human chondrocytes [76]. Moreover we observed a low level, yet sustained activation of ERK1/2. The magnitude and duration of ERK activation may determine the cellular response in many cell types: a high magnitude, acute and transient (minutes) activation as by epidermal growth factor generally induces cell proliferation whereas a low magnitude and prolonged activation (hours) induces differentiation [23,77,78]. Apparently this stands also true for chondrocytic phenotypes, as connective tissue growth factor (CCN2), an agonist that promotes proliferation in de-differentiating rabbit chondrocytes, induces a transient [79] and drastic, over 15fold activation of ERK [40]. A similar ERK1/2 activation profile mediates LPA-induced rat chrondrocyte proliferation [72]. In the latter paradigm, inhibition of another MAPK, p38, did not suppress proliferation, a result also shown for human chondrosarcoma HCS-2/8 and rabbit growth plate cartilage cells treated with CTGF/CCN2, where, however, p38 inhibition induced ^35^[S]-sulfate incorporation, a measure of differentiation [80]. In our studies we did not detect p38 or JNK activation even in short time frames, which we attribute to the fact that PKCε was directly activated with ψεRACK, without the modulation of upstream effectors and induction of cross-talk amongst major signalling pathways that membrane receptor agonism induces. Downstream to PKCε activation, however, different effectors, activated at different time frames may contribute to ERK1/2 activation, as established in neuronal cell types where PKCε may, in addition to Raf, activate FGFR to start a new cycle of ERK activation [24]. Interestingly activation of FGFR in chondrocytes leads to an acute activation of SOX9, which, like in our case, induces aggrecan but not collagen type II expression [81]. 

 In additional support for a differentiation-driving signalling of PKCε in the long term, ψεRACK-dependent activation of ERK led to activation of the CREB1 and AP-1 transcription factors, as postulated with increased synthesis of CREB1 and of the AP-1 homo- and hetero-dimer forming ATF and Fos. This synthesis was completely inhibited by PD98059 and U0126 that prevent activation of MEK1/2 upstream activator of ERK1/2, whereas the selective p38 inhibitor SB203580 had no effect. Therefore the biological effects that we observed may be attributed to ERK1/2 activation, at least amongst MAPKs. Similar magnitude and duration profiles of ERK activation mediate both granulin-epithelin precursor-induced chondrogenesis in human C28I2 chondrocytes [82] and BMP-2 induced transcription of GlcAT-1, a key enzyme for chondroitin sulfate biosynthesis and glycosaminoglycan accumulation in rat NP cells, via AP-1 [83]. Moreover, long-term (40 hours) ψεRACK caused a sustained upregulation of aggrecan expression with concurrent reduction of the proliferation marker Ki67. The initial increase of Ki67 prior to its silencing may feasibly indicate that NP cells first underwent one cell cycle and then proliferation ceased as differentiation prevailed. Similarly, long-term treatment with the pan-PKC activator phorbol ester TPA of rat NP cells in culture resulted first in gains in proliferation and then upregulation of aggrecan expression [26]. This is a common theme and often cells that are induced towards differentiation may first undergo one cell cycle, during which expression of differentiation markers is gained [55,84]. Taken together these data suggest that in response to PKCε activation NP cells entered differentiation in the expense of proliferation. 

 The notion that PKCε activation may regulate aggrecan homeostasis as part of a transcriptional program for differentiation was supported by our findings that ψεRACK in a coordinated manner suppressed the expression of *ADAMTS5*, functionally verified by the reduced cleavage of aggrecan, and increased expression of hsa-mir-377. MicroRNAs have been shown to participate in gene cohort regulation for the implementation of differentiation transcriptional programs in response to PKC in many cell types including chondrocytes [85,86] and chondrogenic differentiation of mesenchymal cells [87]. Our experiments verify the *in silico* analysis that predicted at least 6 possible binding areas for hsa-miR-377 on *ADAMTS5* 3’-UTR and imply that these interactions may have a biological significance on NP cells cartilage homeostasis. This particular microRNA is highly and preferentially expressed in chondrocytes [88], no other regulation, however, of its expression has been reported until now. 

**Figure 7:**
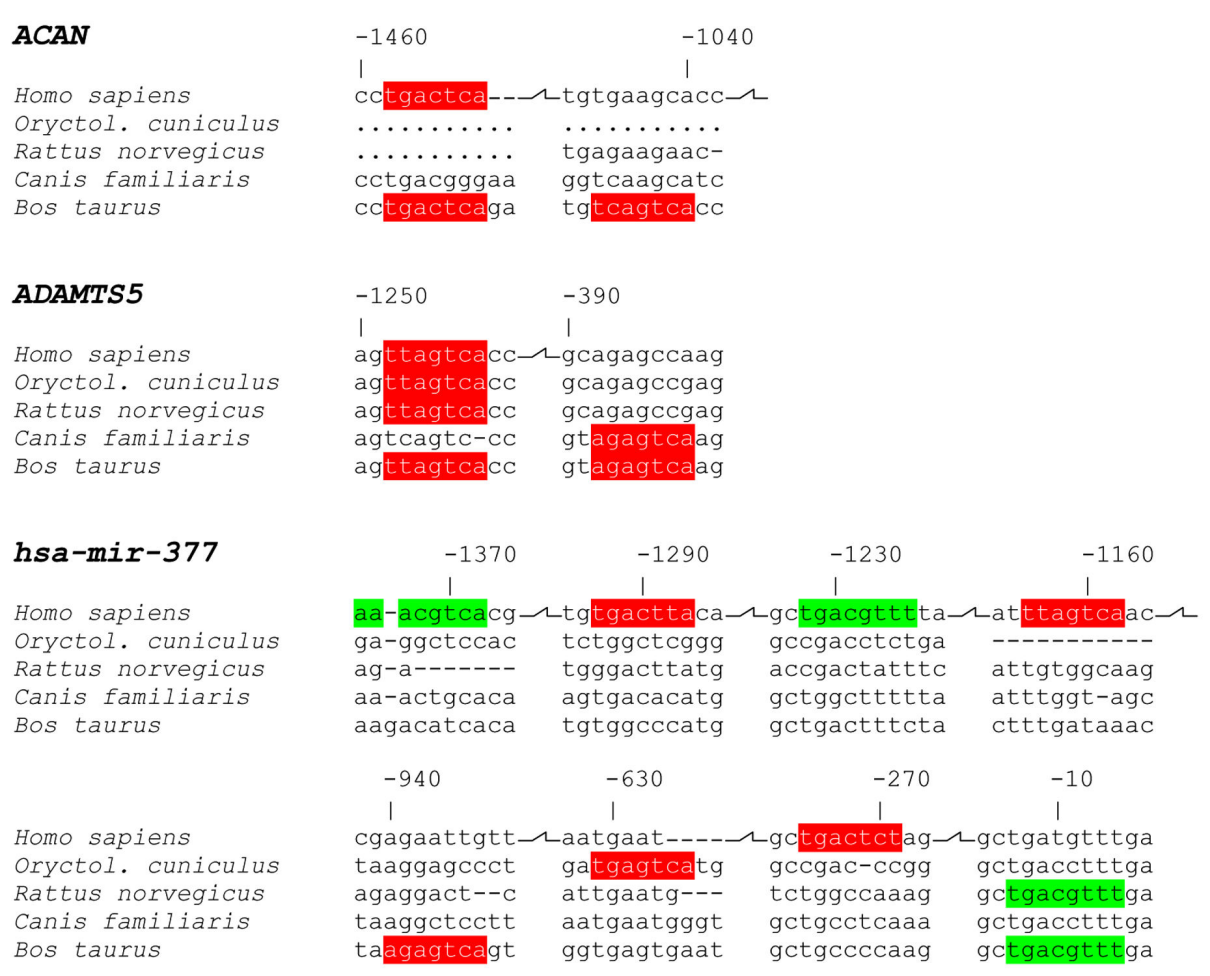
Predicted AP1 and CREB1 binding sites in the ACAN, ADAMTS5, and hsa-mir-377 promoters. Genomic pairwise sequence alignments which correspond to the region that spans 1500bp upstream of the human transcription start site of ACAN and ADAMTS5, or the intergenic region (1375bp) between hsa-mir-377 and its 5’ adjacent hsa-mir-496 were used to identify potential AP1 (red) and CREB1 (green) transcription binding sites. Comparative analysis with four other animal species, namely Oryctolagus cuniculus-rabbit, Rattus norvegicus-rat, Canis familiaris-dog, and Bos taurus-cow that are commonly used for IVD studies, revealed that the ACAN and ADAMTS5 promoters in Homo sapiens are less responsive to AP-1 and CREB activation than the bovine ones, in contrast to the hsa-mir-377 promoter which is enriched in these sites and thus expected to yield higher stoichiometry responses (“--” indicate non available alignments).

 Further support that hsa-miR-377 may be part of a differentiation program came from bioinformatics genome analyses, when we examined AP-1 and CREB1 sites on *ACAN* and *ADAMTS5* promoter sequences and *hsa-miR-377* intergenic region (Figure 7). Apparently, while an AP-1 site at -1458 to -1452 bp from the *ACAN* transcription start site is conserved among Homo sapiens and Bos taurus, a second one is not. A comparable “loss” of AP-1 regulation is detected for human *ADAMTS5*, where the relatively distant from the transcription start site is conserved amongst human, rabbit, rat and cow, but the closer one is lost. In contrast, we found clear gains in CREB1 and AP-1 sites for human miR-377. Specifically, we identified two CREB1 sites as opposed to only one in cow and rat, and an advantage of three sites for AP-1 as compared to only one in cow and rabbit. Taken together with our signalling results, these data suggest that the net outcome of PKCε-dependent activation of AP-1 and CREB1 leads to a clear gain in the expression of the chondrocytic hsa-miR-377, as part of a program that also supports gains in aggrecan expression. 

 Summarizing, in dissecting the molecular basis of availability in human NP cells, our results suggest that PKCε signaling upregulates aggrecan as part of an PKCε/ERK/CREB/AP-1-dependent transcriptional program that includes concurrent upregulation of *ACAN* and hsa-miR-377, and dowregulation of the hsa-miR-377 target *ADAMTS5*. Thus regulation of PKCε and hsa-miR-377, should be considered as novel tools for studies aiming at delineating further IVD degeneration-prone genotypes and at therapeutic restoration of degenerated disks. 

## Supporting Information

Figure S1 **PKCε activation regulates SOX9 expression levels in human NP cells.** When NP cultures were incubated for times indicated with the specific PKCε activator ψεRACK (1µM), a significant increase in SOX9 levels was detected by RT-PCR; the induction lasted for 2 hours and then SOX9 mRNA levels fluctuated back to baseline. SOX9 levels were examined using identical amounts of cDNA template, as for the housekeeping GAPDH (shown at the bottom row); example shown is from three pooled culture dishes of NP7 P8.
(DOCX)

Figure S2 **PKCε activation regulates *ACAN* and *ADAMTS5* expression through ERK activation in human NP cells.** The significant increases in *ACAN* and decreases in *ADAMTS5* expression established by 18 h of incubation with the specific PKCε activator ψεRACK (1µM), were reversed by the MEK1/2 (and therefore ERK1/2) inhibitor U0126. *ACAN* and *ADAMTS5* levels were examined using identical amounts of cDNA template, as for the housekeeping *GAPDH* (shown at the bottom row). Similar results were obtained in three different NP cell lines; examples shown are from three pooled culture dishes of NP8 P13.
(DOCX)

## References

[1] Kalb S, Martirosyan NL, Kalani MY, Broc GG, Theodore N (2012) Genetics of the degenerated intervertebral disc. World Neurosurg 77: 491-501. doi:10.1016/j.wneu.2011.07.014. PubMed: 22120330. 22120330

[2] Antoniou J, Steffen T, Nelson F, Winterbottom N, Hollander AP et al. (1996) The human lumbar intervertebral disc: evidence for changes in the biosynthesis and denaturation of the extracellular matrix with growth, maturation, ageing, and degeneration. J Clin Invest 98: 996-1003. doi:10.1172/JCI118884. PubMed: 8770872. 8770872 PMC507515

[3] Sakai D, Mochida J, Yamamoto Y, Nomura T, Okuma M et al. (2003) Transplantation of mesenchymal stem cells embedded in Atelocollagen gel to the intervertebral disc: a potential therapeutic model for disc degeneration. Biomaterials 24: 3531-3541. doi:10.1016/S0142-9612(03)00222-9. PubMed: 12809782. 12809782

[4] Iwashina T, Mochida J, Sakai D, Yamamoto Y, Miyazaki T et al. (2006) Feasibility of using a human nucleus pulposus cell line as a cell source in cell transplantation therapy for intervertebral disc degeneration. Spine (Phila Pa 1976) 31: 1177-1186. PubMed: 16688029. 10.1097/01.brs.0000217687.36874.c4 16688029

[5] Ganey T, Hutton WC, Moseley T, Hedrick M, Meisel HJ (2009) Intervertebral disc repair using adipose tissue-derived stem and regenerative cells: experiments in a canine model. Spine (Phila Pa 1976) 34: 2297-2304. 10.1097/BRS.0b013e3181a54157 19934809

[6] Miyamoto T, Muneta T, Tabuchi T, Matsumoto K, Saito H et al. (2010) Intradiscal transplantation of synovial mesenchymal stem cells prevents intervertebral disc degeneration through suppression of matrix metalloproteinase-related genes in nucleus pulposus cells in rabbits. Arthritis Res Ther 12: R206. doi:10.1186/ar2952. PubMed: 21054867. 21054867 PMC3046513

[7] Abbott RD, Purmessur D, Monsey RD, Iatridis JC (2012) Regenerative potential of TGFbeta3 + Dex and notochordal cell conditioned media on degenerated human intervertebral disc cells. J Orthop Res 30: 482-488. doi:10.1002/jor.21534. PubMed: 21866573. 21866573 PMC3264846

[8] Kim KW, Ha KY, Lee JS, Nam SW, Woo YK et al. (2009) Notochordal cells stimulate migration of cartilage end plate chondrocytes of the intervertebral disc in in vitro cell migration assays. Spine J 9: 323-329. doi:10.1016/j.spinee.2008.05.003. PubMed: 18619909. 18619909

[9] Minogue BM, Richardson SM, Zeef LA, Freemont AJ, Hoyland JA (2010) Characterization of the human nucleus pulposus cell phenotype and evaluation of novel marker gene expression to define adult stem cell differentiation. Arthritis Rheum 62: 3695-3705. doi:10.1002/art.27710. PubMed: 20722018. 20722018

[10] Sive JI, Baird P, Jeziorsk M, Watkins A, Hoyland JA et al. (2002) Expression of chondrocyte markers by cells of normal and degenerate intervertebral discs. Mol Pathol 55: 91-97. doi:10.1136/mp.55.2.91. PubMed: 11950957. 11950957 PMC1187156

[11] Kluba T, Niemeyer T, Gaissmaier C, Gründer T (2005) Human anulus fibrosis and nucleus pulposus cells of the intervertebral disc: effect of degeneration and culture system on cell phenotype. Spine (Phila Pa 1976) 30: 2743-2748. PubMed: 16371897. 10.1097/01.brs.0000192204.89160.6d 16371897

[12] Aguiar DJ, Johnson SL, Oegema TR (1999) Notochordal cells interact with nucleus pulposus cells: regulation of proteoglycan synthesis. Exp Cell Res 246: 129-137. doi:10.1006/excr.1998.4287. PubMed: 9882522. 9882522

[13] Hunter CJ, Matyas JR, Duncan NA (2003) The notochordal cell in the nucleus pulposus: a review in the context of tissue engineering. Tissue Eng 9: 667-677. doi:10.1089/107632703768247368. PubMed: 13678445. 13678445

[14] Fosang AJ, Rogerson FM, East CJ, Stanton H (2008) ADAMTS-5: the story so far. Eur Cell Mater 15: 11-26. PubMed: 18247274. 18247274 10.22203/ecm.v015a02

[15] Wang J, Markova D, Anderson DG, Zheng Z, Shapiro IM et al. (2011) TNF-alpha and IL-1beta promote a disintegrin-like and metalloprotease with thrombospondin type I motif-5-mediated aggrecan degradation through syndecan-4 in intervertebral disc. J Biol Chem 286: 39738-39749. doi:10.1074/jbc.M111.264549. PubMed: 21949132. 21949132 PMC3220535

[16] Feng G, Li L, Liu H, Song Y, Huang F et al. (2013) Hypoxia differentially regulates human nucleus pulposus and annulus fibrosus cell extracellular matrix production in 3D scaffolds. Osteoarthritis Cartilage 21: 582-588. doi:10.1016/j.joca.2013.01.001. PubMed: 23313531. 23313531

[17] Preradovic A, Kleinpeter G, Feichtinger H, Balaun E, Krugluger W (2005) Quantitation of collagen I, collagen II and aggrecan mRNA and expression of the corresponding proteins in human nucleus pulposus cells in monolayer cultures. Cell Tissue Res 321: 459-464. doi:10.1007/s00441-005-1116-6. PubMed: 16001263. 16001263

[18] Strassburg S, Richardson SM, Freemont AJ, Hoyland JA (2010) Co-culture induces mesenchymal stem cell differentiation and modulation of the degenerate human nucleus pulposus cell phenotype. Regen Med 5: 701-711. doi:10.2217/rme.10.59. PubMed: 20868326. 20868326

[19] Watanabe T, Sakai D, Yamamoto Y, Iwashina T, Serigano K et al. (2010) Human nucleus pulposus cells significantly enhanced biological properties in a coculture system with direct cell-to-cell contact with autologous mesenchymal stem cells. J Orthop Res 28: 623-630. PubMed: 19953600. 19953600 10.1002/jor.21036

[20] Yang SH, Wu CC, Shih TT, Sun YH, Lin FH (2008) In vitro study on interaction between human nucleus pulposus cells and mesenchymal stem cells through paracrine stimulation. Spine (Phila Pa 1976) 33: 1951-1957. 10.1097/BRS.0b013e31817e6974 18708927

[21] Liang W, Ye D, Dai L, Shen Y, Xu J (2012) Overexpression of hTERT extends replicative capacity of human nucleus pulposus cells, and protects against serum starvation-induced apoptosis and cell cycle arrest. J Cell Biochem 113: 2112-2121. doi:10.1002/jcb.24082. PubMed: 22298321. 22298321

[22] Mangoura D, Sun Y, Li C, Singh D, Gutmann DH et al. (2006) Phosphorylation of neurofibromin by PKC is a possible molecular switch in EGF receptor signaling in neural cells. Oncogene 25: 735-745. doi:10.1038/sj.onc.1209113. PubMed: 16314845. 16314845

[23] Leondaritis G, Petrikkos L, Mangoura D (2009) Regulation of the Ras-GTPase activating protein neurofibromin by C-tail phosphorylation: implications for protein kinase C/Ras/extracellular signal-regulated kinase 1/2 pathway signaling and neuronal differentiation. J Neurochem 109: 573-583. doi:10.1111/j.1471-4159.2009.05975.x. PubMed: 19220708. 19220708

[24] Asimaki O, Mangoura D (2011) Cannabinoid receptor 1 induces a biphasic ERK activation via multiprotein signaling complex formation of proximal kinases PKCepsilon, Src, and Fyn in primary neurons. Neurochem Int 58: 135-144. doi:10.1016/j.neuint.2010.11.002. PubMed: 21074588. 21074588

[25] Zisopoulou S, Asimaki O, Leondaritis G, Vasilaki A, Sakellaridis N et al. (2013) PKC-epsilon activation is required for recognition memory in the rat. Behav Brain Res 253: 280-289. doi:10.1016/j.bbr.2013.07.036. PubMed: 23911427. 23911427

[26] Arai F, Hiyama A, Sakai D, Yokoyama K, Mochida J (2012) The expression and role of non-canonical (PKC) signaling in nucleus pulposus cell metabolism. J Orthop Res 30: 1478-1485. doi:10.1002/jor.22095. PubMed: 22389031. 22389031

[27] Ding L, Guo D, Homandberg GA (2009) Fibronectin fragments mediate matrix metalloproteinase upregulation and cartilage damage through proline rich tyrosine kinase 2, c-src, NF-kappaB and protein kinase Cdelta. Osteoarthritis Cartilage 17: 1385-1392. doi:10.1016/j.joca.2009.03.024. PubMed: 19409294. 19409294

[28] Xia M, Zhu Y (2011) Fibronectin fragment activation of ERK increasing integrin alpha(5) and beta(1) subunit expression to degenerate nucleus pulposus cells. J Orthop Res 29: 556-561. doi:10.1002/jor.21273. PubMed: 21337395. 21337395

[29] Yoon YM, Oh CD, Kang SS, Chun JS (2000) Protein kinase A regulates chondrogenesis of mesenchymal cells at the post-precartilage condensation stage via protein kinase C-alpha signaling. J Bone Miner Res 15: 2197-2205. doi:10.1359/jbmr.2000.15.11.2197. PubMed: 11092400. 11092400

[30] Friedman RC, Farh KK, Burge CB, Bartel DP (2009) Most mammalian mRNAs are conserved targets of microRNAs. Genome Res 19: 92-105. PubMed: 18955434. 18955434 10.1101/gr.082701.108 PMC2612969

[31] Artmann S, Jung K, Bleckmann A, Beissbarth T (2012) Detection of simultaneous group effects in microRNA expression and related target gene sets. PLOS ONE 7: e38365. doi:10.1371/journal.pone.0038365. PubMed: 22723856. 22723856 PMC3378551

[32] Baskerville S, Bartel DP (2005) Microarray profiling of microRNAs reveals frequent coexpression with neighboring miRNAs and host genes. RNA 11: 241-247. doi:10.1261/rna.7240905. PubMed: 15701730. 15701730 PMC1370713

[33] Jayawardena TM, Egemnazarov B, Finch EA, Zhang L, Payne JA et al. (2012) MicroRNA-mediated in vitro and in vivo direct reprogramming of cardiac fibroblasts to cardiomyocytes. Circ Res 110: 1465-1473. doi:10.1161/CIRCRESAHA.112.269035. PubMed: 22539765. 22539765 PMC3380624

[34] Schwartz Z, Ehland H, Sylvia VL, Larsson D, Hardin RR et al. (2002) 1alpha,25-dihydroxyvitamin D(3) and 24R,25-dihydroxyvitamin D(3) modulate growth plate chondrocyte physiology via protein kinase C-dependent phosphorylation of extracellular signal-regulated kinase 1/2 mitogen-activated protein kinase. Endocrinology 143: 2775-2786. doi:10.1210/en.143.7.2775. PubMed: 12072413. 12072413

[35] Brandman R, Disatnik MH, Churchill E, Mochly-Rosen D (2007) Peptides derived from the C2 domain of protein kinase C epsilon (epsilon PKC) modulate epsilon PKC activity and identify potential protein-protein interaction surfaces. J Biol Chem 282: 4113-4123. PubMed: 17142835. 17142835 10.1074/jbc.M608521200

[36] Bright R, Sun GH, Yenari MA, Steinberg GK, Mochly-Rosen D (2008) epsilonPKC confers acute tolerance to cerebral ischemic reperfusion injury. Neurosci Lett 441: 120-124. doi:10.1016/j.neulet.2008.05.080. PubMed: 18586397. 18586397 PMC2597630

[37] Qvit N, Mochly-Rosen D (2010) Highly Specific Modulators of Protein Kinase C Localization: Applications to Heart Failure. Drug Discov Today Dis Mech 7: e87-e93. doi:10.1016/j.ddmec.2010.07.001. PubMed: 21151743. 21151743 PMC2998291

[38] Kim SJ, Chun JS (2003) Protein kinase C alpha and zeta regulate nitric oxide-induced NF-kappa B activation that mediates cyclooxygenase-2 expression and apoptosis but not dedifferentiation in articular chondrocytes. Biochem Biophys Res Commun 303: 206-211. doi:10.1016/S0006-291X(03)00305-X. PubMed: 12646188. 12646188

[39] Peng H, Zhou JL, Liu SQ, Hu QJ, Ming JH et al. (2010) Hyaluronic acid inhibits nitric oxide-induced apoptosis and dedifferentiation of articular chondrocytes in vitro. Inflamm Res 59: 519-530. doi:10.1007/s00011-010-0156-x. PubMed: 20077126. 20077126

[40] Yoon YM, Kim SJ, Oh CD, Ju JW, Song WK et al. (2002) Maintenance of differentiated phenotype of articular chondrocytes by protein kinase C and extracellular signal-regulated protein kinase. J Biol Chem 277: 8412-8420. doi:10.1074/jbc.M110608200. PubMed: 11744731. 11744731

[41] Lee YA, Kang SS, Baek SH, Jung JC, Jin EJ et al. (2007) Redifferentiation of dedifferentiated chondrocytes on chitosan membranes and involvement of PKCalpha and P38 MAP kinase. Mol Cells 24: 9-15. PubMed: 17846494. 17846494

[42] LaVallie ER, Chockalingam PS, Collins-Racie LA, Freeman BA, Keohan CC et al. (2006) Protein kinase Czeta is up-regulated in osteoarthritic cartilage and is required for activation of NF-kappaB by tumor necrosis factor and interleukin-1 in articular chondrocytes. J Biol Chem 281: 24124-24137. doi:10.1074/jbc.M601905200. PubMed: 16798739. 16798739

[43] Chockalingam PS, Varadarajan U, Sheldon R, Fortier E, LaVallie ER et al. (2007) Involvement of protein kinase Czeta in interleukin-1beta induction of ADAMTS-4 and type 2 nitric oxide synthase via NF-kappaB signaling in primary human osteoarthritic chondrocytes. Arthritis Rheum 56: 4074-4083. doi:10.1002/art.23043. PubMed: 18050214. 18050214

[44] Litherland GJ, Elias MS, Hui W, Macdonald CD, Catterall JB et al. (2010) Protein kinase C isoforms zeta and iota mediate collagenase expression and cartilage destruction via STAT3- and ERK-dependent c-fos induction. J Biol Chem 285: 22414-22425. doi:10.1074/jbc.M110.120121. PubMed: 20463008. 20463008 PMC2903406

[45] DelCarlo M, Loeser RF (2006) Chondrocyte cell death mediated by reactive oxygen species-dependent activation of PKC-betaI. Am J Physiol Cell Physiol 290: C802-C811. PubMed: 16236825. 16236825 10.1152/ajpcell.00214.2005 PMC1482466

[46] Im HJ, Muddasani P, Natarajan V, Schmid TM, Block JA et al. (2007) Basic fibroblast growth factor stimulates matrix metalloproteinase-13 via the molecular cross-talk between the mitogen-activated protein kinases and protein kinase Cdelta pathways in human adult articular chondrocytes. J Biol Chem 282: 11110-11121. doi:10.1074/jbc.M609040200. PubMed: 17311929. 17311929 PMC2895271

[47] Campo GM, Avenoso A, Campo S, D'Ascola A, Traina P et al. (2009) Differential effect of molecular size HA in mouse chondrocytes stimulated with PMA. Biochim Biophys Acta 1790: 1353-1367. doi:10.1016/j.bbagen.2009.07.003. PubMed: 19607883. 19607883

[48] Yoon YM, Oh CD, Kim DY, Lee YS, Park JW et al. (2000) Epidermal growth factor negatively regulates chondrogenesis of mesenchymal cells by modulating the protein kinase C-alpha, Erk-1, and p38 MAPK signaling pathways. J Biol Chem 275: 12353-12359. doi:10.1074/jbc.275.16.12353. PubMed: 10766877. 10766877

[49] Kawata K, Kubota S, Eguchi T, Moritani NH, Shimo T et al. (2010) Role of the low-density lipoprotein receptor-related protein-1 in regulation of chondrocyte differentiation. J Cell Physiol 222: 138-148. doi:10.1002/jcp.21930. PubMed: 19795391. 19795391

[50] Shapiro IM, Leboy PS, Tokuoka T, Forbes E, DeBolt K et al. (1991) Ascorbic acid regulates multiple metabolic activities of cartilage cells. Am J Clin Nutr 54: 1209S-1213S. PubMed: 1962572. 1962572 10.1093/ajcn/54.6.1209s

[51] Peterson L, Brittberg M, Kiviranta I, Akerlund EL, Lindahl A (2002) Autologous chondrocyte transplantation. Biomechanics and long-term durability. Am J Sports Med 30: 2-12. PubMed: 11798989. 11798989 10.1177/03635465020300011601

[52] Schwartz NB, Domowicz M, Krueger RC Jr., Li H, Mangoura D (1996) Brain aggrecan. Perspect Dev Neurobiol 3: 291-306. PubMed: 9117261. 9117261

[53] Domowicz MS, Mangoura D, Schwartz NB (2003) Aggrecan regulates telencephalic neuronal aggregation in culture. Brain Res. Dev Brain Res 143: 207-216. doi:10.1016/S0165-3806(03)00133-0. 12855192

[54] Hennig AK, Mangoura D, Schwartz NB (1993) Large chondroitin sulfate proteoglycans of developing chick CNS are expressed in cerebral hemisphere neuronal cultures. Brain Res. Dev Brain Res 73: 261-272. doi:10.1016/0165-3806(93)90146-2. 8353936

[55] Mangoura D, Pelletiere C, Leung S, Sakellaridis N, Wang DX (2000) Prolactin concurrently activates src-PLD and JAK/Stat signaling pathways to induce proliferation while promoting differentiation in embryonic astrocytes. Int J Dev Neurosci 18: 693-704. doi:10.1016/S0736-5748(00)00031-9. PubMed: 10978848. 10978848

[56] McLaughlin D, Tsirimonaki E, Vallianatos G, Sakellaridis N, Chatzistamatiou T et al. (2006) Stable expression of a neuronal dopaminergic progenitor phenotype in cell lines derived from human amniotic fluid cells. J Neurosci Res 83: 1190-1200. doi:10.1002/jnr.20828. PubMed: 16555279. 16555279

[57] Oshevski S, Le Bousse-Kerdilès MC, Clay D, Levashova Z, Debili N et al. (1999) Differential expression of protein kinase C isoform transcripts in human hematopoietic progenitors undergoing differentiation. Biochem Biophys Res Commun 263: 603-609. doi:10.1006/bbrc.1999.1425. PubMed: 10512725. 10512725

[58] Broos S, Hulpiau P, Galle J, Hooghe B, Van Roy F et al. (2011) ConTra v2: a tool to identify transcription factor binding sites across species, update 2011. Nucleic Acids Res 39: W74-W78. doi:10.1093/nar/gkr130. PubMed: 21576231. 21576231 PMC3125763

[59] Flicek P, Amode MR, Barrell D, Beal K, Brent S et al. (2012) Ensembl 2012. Nucleic Acids Res 40: D84-D90. doi:10.1093/nar/gks210. PubMed: 22086963. 22086963 PMC3245178

[60] Kel AE, Gössling E, Reuter I, Cheremushkin E, Kel-Margoulis OV et al. (2003) MATCH: A tool for searching transcription factor binding sites in DNA sequences. Nucleic Acids Res 31: 3576-3579. doi:10.1093/nar/gkg585. PubMed: 12824369. 12824369 PMC169193

[61] Logotheti S, Papaevangeliou D, Michalopoulos I, Sideridou M, Tsimaratou K et al. (2012) Progression of mouse skin carcinogenesis is associated with increased eralpha levels and is repressed by a dominant negative form of eralpha. PLOS ONE 7: e41957. doi:10.1371/journal.pone.0041957. PubMed: 22870269. 22870269 PMC3411716

[62] Portales-Casamar E, Thongjuea S, Kwon AT, Arenillas D, Zhao X et al. (2010) JASPAR 2010: the greatly expanded open-access database of transcription factor binding profiles. Nucleic Acids Res 38: D105-D110. doi:10.1093/nar/gkp950. PubMed: 19906716. 19906716 PMC2808906

[63] Gan JC, Ducheyne P, Vresilovic EJ, Shapiro IM (2003) Intervertebral disc tissue engineering II: cultures of nucleus pulposus cells. Clin Orthop Relat Res: 315-324. 10.1097/01.blo.0000063797.98363.d3 12782890

[64] Groma G, Grskovic I, Schael S, Ehlen HW, Wagener R et al. (2011) Matrilin-4 is processed by ADAMTS-5 in late Golgi vesicles present in growth plate chondrocytes of defined differentiation state. Matrix Biol 30: 275-280. doi:10.1016/j.matbio.2011.04.002. PubMed: 21539915. 21539915

[65] Pockert AJ, Richardson SM, Le Maitre CL, Lyon M, Deakin JA et al. (2009) Modified expression of the ADAMTS enzymes and tissue inhibitor of metalloproteinases 3 during human intervertebral disc degeneration. Arthritis Rheum 60: 482-491. doi:10.1002/art.24291. PubMed: 19180493. 19180493

[66] Struglics A, Lohmander LS, Last K, Akikusa J, Allen R et al. (2012) Aggrecanase cleavage in juvenile idiopathic arthritis patients is minimally detected in the aggrecan interglobular domain but robust at the aggrecan C-terminus. Arthritis Rheum 64: 4151-4162; author reply: 22886575. 22886575 10.1002/art.34665

[67] Wu L, Liu J, Gao P, Nakamura M, Cao Y et al. (2005) Transforming activity of MECT1-MAML2 fusion oncoprotein is mediated by constitutive CREB activation. EMBO J 24: 2391-2402. doi:10.1038/sj.emboj.7600719. PubMed: 15961999. 15961999 PMC1173159

[68] Risbud MV, Fertala J, Vresilovic EJ, Albert TJ, Shapiro IM (2005) Nucleus pulposus cells upregulate PI3K/Akt and MEK/ERK signaling pathways under hypoxic conditions and resist apoptosis induced by serum withdrawal. Spine (Phila Pa 1976) 30: 882-889. PubMed: 15834331. 10.1097/01.brs.0000159096.11248.6d 15834331

[69] Rastogi A, Thakore P, Leung A, Benavides M, Machado M et al. (2009) Environmental regulation of notochordal gene expression in nucleus pulposus cells. J Cell Physiol 220: 698-705. doi:10.1002/jcp.21816. PubMed: 19472213. 19472213

[70] Barrionuevo F, Taketo MM, Scherer G, Kispert A (2006) Sox9 is required for notochord maintenance in mice. Dev Biol 295: 128-140. doi:10.1016/j.ydbio.2006.03.014. PubMed: 16678811. 16678811

[71] Akiyama H, Lefebvre V (2011) Unraveling the transcriptional regulatory machinery in chondrogenesis. J Bone Miner Metab 29: 390-395. doi:10.1007/s00774-011-0273-9. PubMed: 21594584. 21594584 PMC3354916

[72] Kim MK, Lee HY, Park KS, Shin EH, Jo SH et al. (2005) Lysophosphatidic acid stimulates cell proliferation in rat chondrocytes. Biochem Pharmacol 70: 1764-1771. doi:10.1016/j.bcp.2005.09.015. PubMed: 16242672. 16242672

[73] Hurst-Kennedy J, Boyan BD, Schwartz Z (2009) Lysophosphatidic acid signaling promotes proliferation, differentiation, and cell survival in rat growth plate chondrocytes. Biochim Biophys Acta 1793: 836-846. doi:10.1016/j.bbamcr.2009.01.020. PubMed: 19233232. 19233232

[74] Gilson A, Dreger M, Urban JP (2010) Differential expression level of cytokeratin 8 in cells of the bovine nucleus pulposus complicates the search for specific intervertebral disc cell markers. Arthritis Res Ther 12: R24. doi:10.1186/ar2931. PubMed: 20152014. 20152014 PMC2875658

[75] Minogue BM, Richardson SM, Zeef LA, Freemont AJ, Hoyland JA (2010) Transcriptional profiling of bovine intervertebral disc cells: implications for identification of normal and degenerate human intervertebral disc cell phenotypes. Arthritis Res Ther 12: R22. doi:10.1186/ar2929. PubMed: 20149220. 20149220 PMC2875656

[76] Andreas K, Häupl T, Lübke C, Ringe J, Morawietz L et al. (2009) Antirheumatic drug response signatures in human chondrocytes: potential molecular targets to stimulate cartilage regeneration. Arthritis Res Ther 11: R15. doi:10.1186/ar2605. PubMed: 19192274. 19192274 PMC2688247

[77] Marshall CJ (1995) Specificity of receptor tyrosine kinase signaling: transient versus sustained extracellular signal-regulated kinase activation. Cell 80: 179-185. doi:10.1016/0092-8674(95)90401-8. PubMed: 7834738. 7834738

[78] Leondaritis G, Koliou X, Johnson S, Li C, Florakis A, et al. (2011) Interplay between Protein kinase C isoforms alpha and epsilon, neurofibromin. In: Shimada H Neuroblastoma Rijeka: Intech . pp. 85-110

[79] Yosimichi G, Kubota S, Nishida T, Kondo S, Yanagita T et al. (2006) Roles of PKC, PI3K and JNK in multiple transduction of CCN2/CTGF signals in chondrocytes. Bone 38: 853-863. doi:10.1016/j.bone.2005.11.016. PubMed: 16431170. 16431170

[80] Yosimichi G, Nakanishi T, Nishida T, Hattori T, Takano-Yamamoto T et al. (2001) CTGF/Hcs24 induces chondrocyte differentiation through a p38 mitogen-activated protein kinase (p38MAPK), and proliferation through a p44/42 MAPK/extracellular-signal regulated kinase (ERK). Eur J Biochem 268: 6058-6065. doi:10.1046/j.0014-2956.2001.02553.x. PubMed: 11732999. 11732999

[81] Murakami S, Kan M, McKeehan WL, de Crombrugghe B (2000) Up-regulation of the chondrogenic Sox9 gene by fibroblast growth factors is mediated by the mitogen-activated protein kinase pathway. Proc Natl Acad Sci U S A 97: 1113-1118. doi:10.1073/pnas.97.3.1113. PubMed: 10655493. 10655493 PMC15539

[82] Feng JQ, Guo FJ, Jiang BC, Zhang Y, Frenkel S et al. (2010) Granulin epithelin precursor: a bone morphogenic protein 2-inducible growth factor that activates Erk1/2 signaling and JunB transcription factor in chondrogenesis. FASEB J 24: 1879-1892. doi:10.1096/fj.09-144659. PubMed: 20124436. 20124436 PMC2874481

[83] Hiyama A, Gogate SS, Gajghate S, Mochida J, Shapiro IM et al. (2010) BMP-2 and TGF-beta stimulate expression of beta1,3-glucuronosyl transferase 1 (GlcAT-1) in nucleus pulposus cells through AP1, TonEBP, and Sp1: role of MAPKs. J Bone Miner Res 25: 1179-1190. PubMed: 19961337. 19961337 10.1359/jbmr.091202 PMC3153993

[84] Cheng Y, Zhizhin I, Perlman RL, Mangoura D (2000) Prolactin-induced cell proliferation in PC12 cells depends on JNK but not ERK activation. J Biol Chem 275: 23326-23332. doi:10.1074/jbc.M001837200. PubMed: 10807911. 10807911

[85] Miyaki S, Nakasa T, Otsuki S, Grogan SP, Higashiyama R et al. (2009) MicroRNA-140 is expressed in differentiated human articular chondrocytes and modulates interleukin-1 responses. Arthritis Rheum 60: 2723-2730. doi:10.1002/art.24745. PubMed: 19714579. 19714579 PMC2806094

[86] Sonkoly E, Wei T, Pavez Loriè E, Suzuki H, Kato M et al. (2010) Protein kinase C-dependent upregulation of miR-203 induces the differentiation of human keratinocytes. J Invest Dermatol 130: 124-134. doi:10.1038/jid.2009.294. PubMed: 19759552. 19759552

[87] Yang B, Guo H, Zhang Y, Chen L, Ying D et al. (2011) MicroRNA-145 regulates chondrogenic differentiation of mesenchymal stem cells by targeting Sox9. PLOS ONE 6: e21679. doi:10.1371/journal.pone.0021679. PubMed: 21799743. 21799743 PMC3140487

[88] Kobayashi T, Lu J, Cobb BS, Rodda SJ, McMahon AP et al. (2008) Dicer-dependent pathways regulate chondrocyte proliferation and differentiation. Proc Natl Acad Sci U S A 105: 1949-1954. doi:10.1073/pnas.0707900105. PubMed: 18238902. 18238902 PMC2538863

