# Supplementary figures and images for: PKCε Signalling Activates ERK1/2, and Regulates Aggrecan, ADAMTS5, and miR377 Gene Expression in Human Nucleus Pulposus Cells
Source: PLoS One. 2013 Nov 28;8(11):e82045. doi: 10.1371/journal.pone.0082045 (PMC3842981; doi:10.1371/journal.pone.0082045)

**
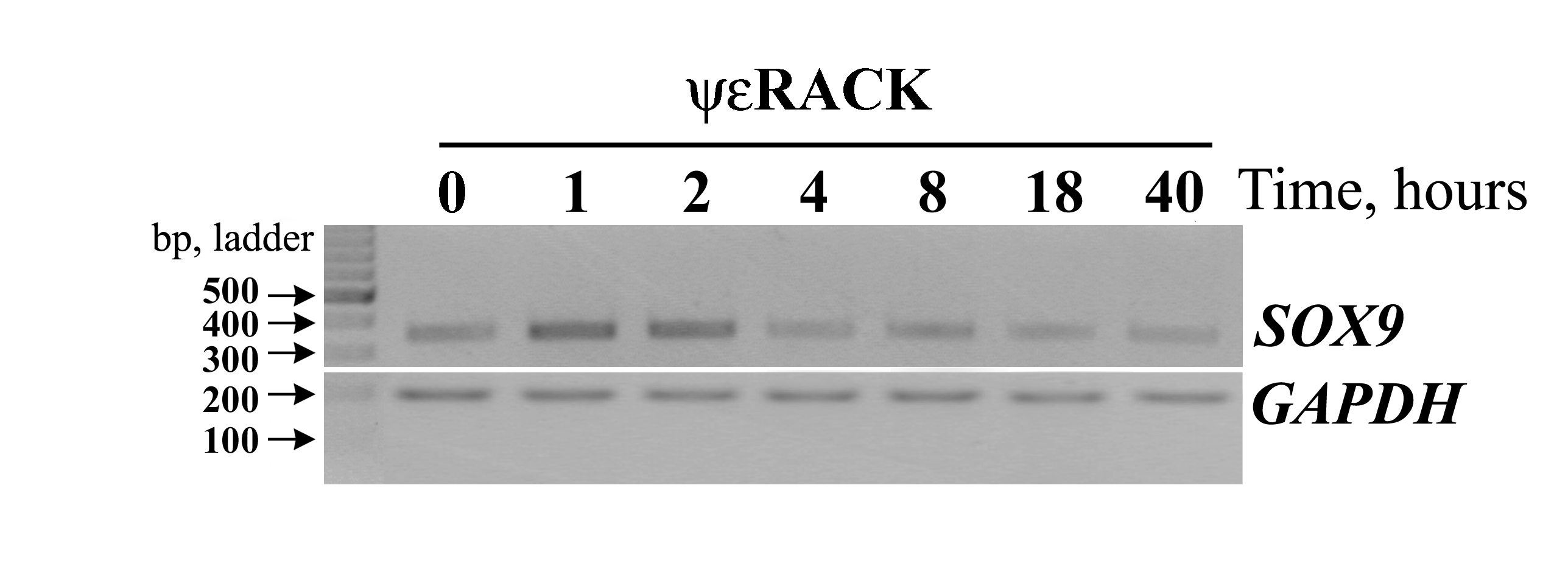
**

Supplement: Figure S1 — PKCε activation regulates SOX9 expression levels in human NP cells. When NP cultures were incubated for times indicated with the specific PKCε activator ψεRACK (1µM), a significant increase in SOX9 levels was detected by RT-PCR; the induction lasted for 2 hours and then SOX9 mRNA levels fluctuated back to baseline. SOX9 levels were examined using identical amounts of cDNA template, as for the housekeeping GAPDH (shown at the bottom row); example shown is from three pooled culture dishes of NP7 P8. (DOCX) [file pone.0082045.s001.docx]
